# Metal-Free α-C(*sp*^3^)–H Functionalized Oxidative Cyclization of Tertiary *N,N*-Diaryl Amino Alcohols: Theoretical Approach for Mechanistic Pathway

**DOI:** 10.3390/molecules22040547

**Published:** 2017-03-29

**Authors:** Zakir Ullah, Mihyun Kim

**Affiliations:** Gachon Institute of Pharmaceutical Science & Department of Pharmacy, College of Pharmacy, Gachon University, Yeonsu-gu, Incheon 21936, Korea; azazay1@gmail.com

**Keywords:** α-C(*sp*^3^)–H functionalization, oxidative cyclization, tertiary amine, 6-*exo*-*trig*, metal-free C–H activation

## Abstract

The mechanistic pathway of TEMPO/I_2_-mediated oxidative cyclization of *N,N*-diaryl amino alcohols **1** was investigated. Based on direct empirical experiments, three key intermediates (aminium radical cation **3**, α-aminoalkyl radical **4**, and iminium **5**), four types of reactive species (radical TEMPO, cationic TEMPO, TEMPO-I, and iodo radical), and three types of pathways ((1) SET/PCET mechanism; (2) HAT/1,6-H transfer mechanism; (3) ionic mechanism) were assumed. Under the assumption, nine free energy diagrams were acquired through density functional theory calculations. From the comparison of solution-phase free energy, some possible mechanisms were excluded, and then the chosen plausible mechanisms were concretized using the more stable intermediate **7**.

## 1. Introduction

Because of reasons like cost and toxicity, metal-free C–H activation has been developed [1]. Although iodine is a nonmetal, it can behave like a transition metal in diverse reactions including ligand exchange, oxidative addition, reductive elimination, and ligand coupling. When a three-center–four-electron (3c–4e) bond (L–I–L) (hypervalent bond) is formed by the overlap of the *5p* orbital on iodine atom with the orbitals on the two ligands L, transition-metal-like properties are observed. Therefore, in most studies, hypervalent iodine(III/V) was utilized for metal-free C–H functionalization. In particular, recently, metal-free cross-dehydrogenative coupling (CDC) reactions were feasible using hypervalent iodine(III) [2,3]. However, compared to the versatile utilization of metal-free reactions, the mechanisms of metal-free reactions have not been extensively investigated, and various plausible hypotheses have been reported for new reaction methodologies. Some studies reported an ionic mechanism, and others reported a SET mechanism [4,5,6].

**Figure 1:**
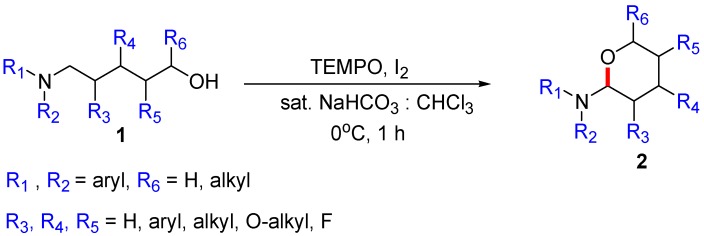
TEMPO/I_2_-mediated oxidative cyclization of *N,N*-diarylamino alcohol **1**.

“TEMPO/BAIB” and “TEMPO/I_2_” are standard reagents for the oxidation of an alcohol to an aldehyde. While several leading researchers reported iodine as a catalyst [7,8], iodine has been widely used as a co-oxidant owing to its low cost and benign byproduct. Recently, our research group published the oxidative cyclization of *N,N*-diarylamino alcohol **1** by the α-C(*sp*^3^)–H functionalization of amines under the condition of Figure 1 [9]. Based on a literature search, we could explain that the CDC reaction resulted from the *tert*-amino effect [10]. However, in the study, we could not answer the following questions: (1) “What is the direct reactive species for the functionalization”? (2) “Which type of intermediate is dominant”? (3) “How does the well-known radical-trapping agent TEMPO contribute to the reaction—as a direct catalyst or precatalyst, radical trapper or initiator/accelerator/controller to prevent the undesired side reactions”?

In this study, three types of pathways were considered: (1) aminium radical cation **3** and SET/PCET mechanism; (2) α-aminoalkyl radical **4** and HAT mechanism; (3) conventional ionic mechanism under basic conditions. Density functional theory (DFT) calculations were carried out to obtain energy diagrams so that a more acceptable pathway from the three mechanisms can be selected. In addition, the dominant reactive species in the three energy diagrams can be proposed.

## 2. Initial Assumptions and Theoretical Background

**Figure 2:**
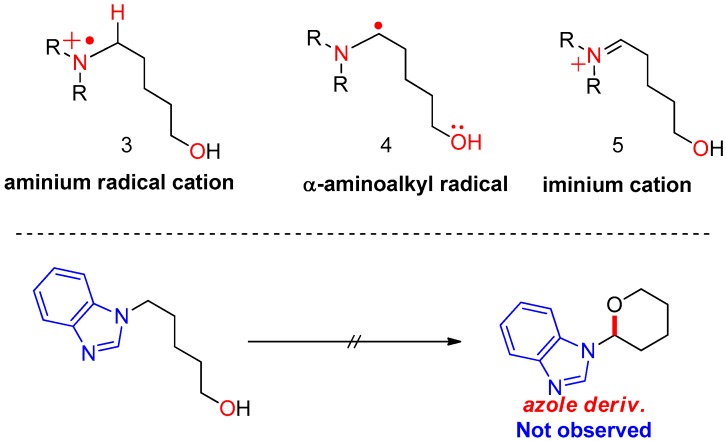
Initially assumed key intermediates of oxidative cyclization.

First, plausible catalytic cycles were considered for the CDC of amino alcohols using TEMPO as the catalyst and iodine as the oxidant/hydrogen acceptor. In our previous study, the reaction of azole derivatives bearing *sp*^2^-hybridized nitrogen (e.g., benzimidazoyl amino alcohol or carbazoyl amino alcohol) did not afford the cyclization product **2** at all; therefore, we proposed that the key intermediate in the reaction is iminium **5**, requiring the loss of aromaticity of the azole ring in azole substrates. In sequence, how to generate the activated iminium **5** was considered. If the reaction follows a nonionic mechanism, the oxidants or reactive species from the oxidants receive an e^−^ (via SET) or H^+^/e^−^ (via radical transfer) from substrate **1**, producing intermediates **3** or **4** shown in Figure 2. In the first case, oxidants or species generated from the oxidants (either TEMPO or BAIB) abstract an electron from the nitrogen of the substrate (via SET), generating aminium radical cation **3**. Here, radical cation **3** arising from the electron transfer should be stable. In the second case, a free radical removes a hydrogen atom from the α-carbon of the substrate, producing α-aminoalkyl radical **4**. The yield of cyclization product **2** depends on the stability of aminium radical cation (R_3_N^•+^) **3** and α-aminoalkyl radical intermediate **4**.

**Figure 3:**
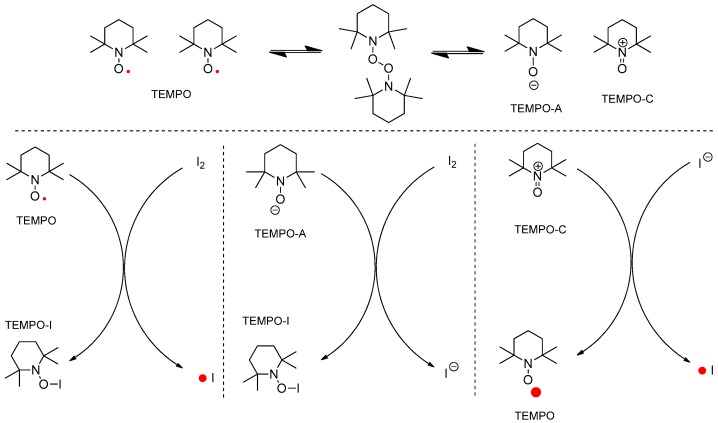
Plausible reactive species generated from TEMPO/I_2_ in nonionic mechanism.

**Figure 4:**
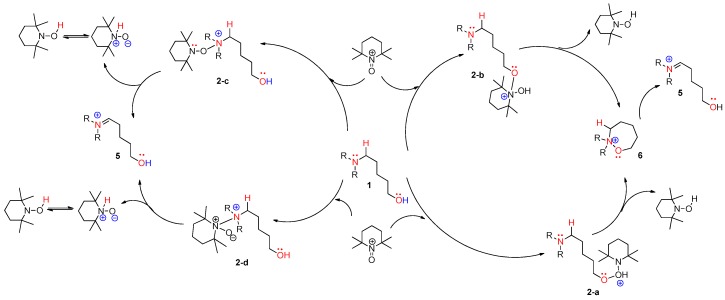
Plausible ionic mechanism of oxidative cyclization.

TEMPO has been determined as the active catalyst for hydrogen transfer in an oxidation reaction and as the radical scavenger in a radical chain reaction [11,12,13,14,15]. In the combination of TEMPO and iodine, the next reactive species were considered to generate intermediates **3** or **4** (two different mechanisms) shown in Figure 3. In addition, the typical regeneration of TEMPO from TEMPO-H was not considered in the ionic mechanism shown in Figure 4.

If the reaction follows an ionic mechanism such as in the typical oxidation of an alcohol to an aldehyde, the next four types of ionic routes can be considered for the ionic mechanism in Figure 4. The left cycle explains the new bond formation between TEMPO and nitrogen, and the right cycle explains the bond formation between TEMPO and the oxygen of the hydroxyl group in substrate **2**. Every “N–N”, “N–O”, and “O–O” bond formation produces activated iminium **5**.

## 3. Computational Methods

All the calculations were carried out using DFT [16] as implemented in the Jaguar 8.0 suite of ab initio quantum chemistry programs [17]. Geometry optimizations were performed using the B3LYP [18,19,20,21,22] functional and 6-31G** basis set. The energies of the optimized structures were re-evaluated by additional single-point energy calculations of each optimized geometry using Dunning’s correlation consistent triple-ζ basis set cc-pVTZ(-f) that includes a double set of polarization functions [23]. Solvation energies were evaluated using the self-consistent reaction field (SCRF) approach based on the accurate numerical solutions of the Poisson–Boltzmann equation [24,25,26]. Solvation calculations were carried out using the 6-31G**/LACVP basis [27,28] at the optimized gas-phase geometry using a dielectric constant of ε = 8.93 for dichloromethane. For all the continuum models, the solvation energies are subject to the empirical parameterization of atomic radii used to generate the solute surface. The standard set of optimized radii was used in Jaguar for H (1.150 Å), B (2.042 Å), C (1.900 Å), N (1.600 Å), O (1.550 Å), P (2.074 Å), and I (2.250 Å). Analytical vibrational frequencies within the harmonic approximation were computed using the 6-31G**/LACVP basis set to confirm proper convergence to well-defined minima or saddle points on the potential energy surface. The electron attachment energy in solution, ∆GEA, was calculated by computing the energy components as follows:

∆GEA(sol) = ∆GEA(GP) + ∆Gsolv
(1)

∆GEA(GP) = ∆HEA(GP) − T∆S(GP)
(2)

∆HEA(GP) = ∆EEA(SCF) + ∆ZPE
(3)

∆GEA(GP) is the free energy in gas phase; ∆Gsolv is the free energy of solvation computed using the continuum solvation model; ∆HEA(GP) is the enthalpy in the gas phase; T is the temperature (298 K); ∆S(GP) is the entropy in the gas phase; ∆EEA(SCF) is the self-consistent field energy, i.e., the “raw” electronic energy computed from the SCF procedure; ZPE is the zero-point energy. The ZPE and entropy were obtained from the vibrational frequency calculation. Note that entropy specifically refers to the vibrational/rotational/translational entropy of the solute(s); the entropy of the solvent was incorporated implicitly in the continuum solvation model.

## 4. Results and Discussion

### 4.1. Pathway A: Aminium Radical Cation 3 and SET/PCET Mechanism

**Figure 5:**
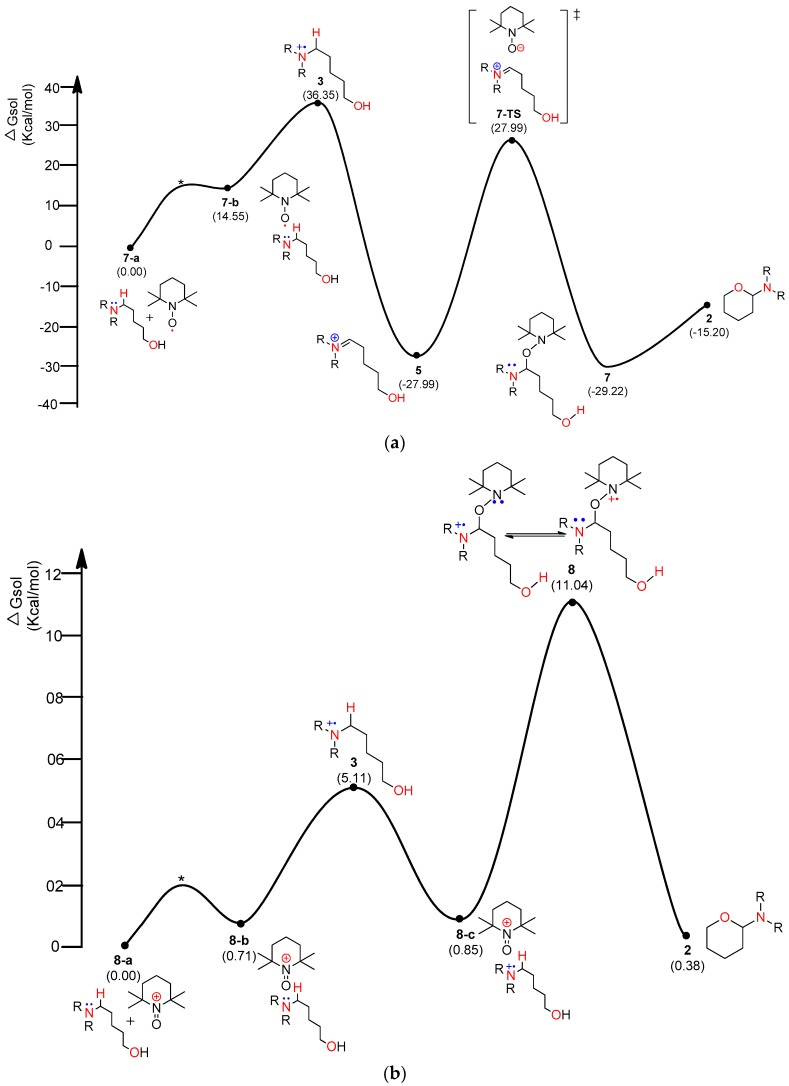

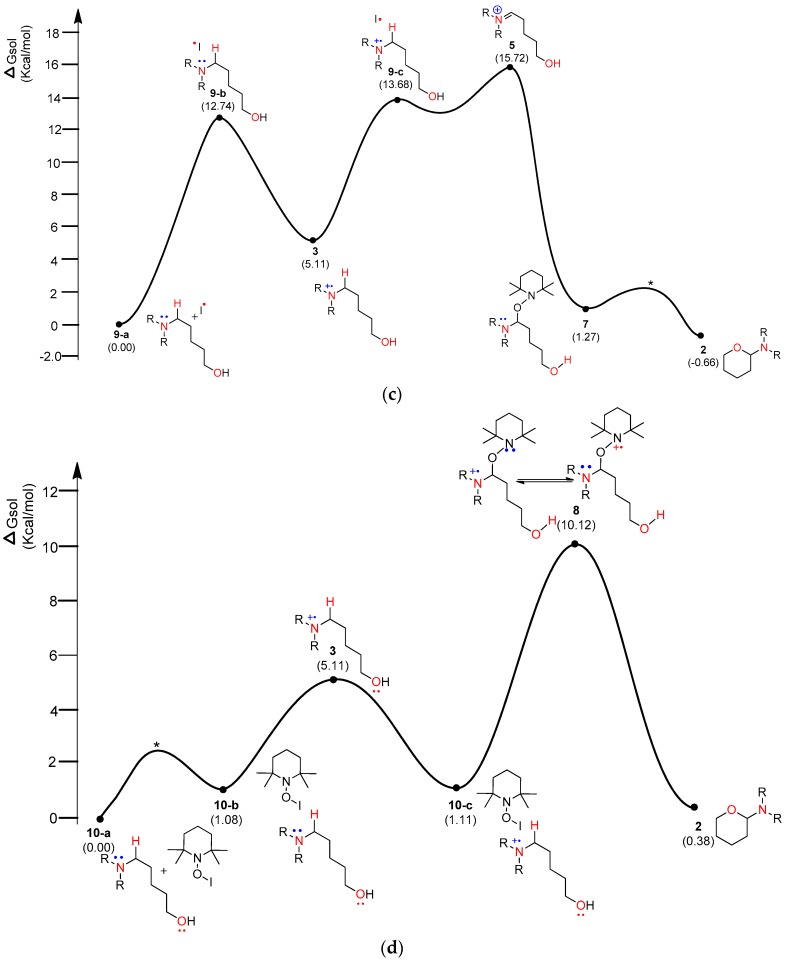
Calculated energy profile diagram for metal-free CDC reaction through pathway A: (**a**) intermediate **3** resulted from TEMPO; (**b**) intermediate **3** resulted from TEMPO-C; (**c**) intermediate **3** resulted from iodine radical; (**d**) intermediate **3** resulted from TEMPO-I; R-group is phenyl; transition state marked by asterisk was not explicitly located. Transition state marked by asterisk (*) were not explicity located.

In general, aminium radical cations (R_3_N^•+^)—particularly from arylamines—are involved in the proton-coupled electron transfer (PCET) reaction of amines to produce iminium species [29,30]. Therefore, homolytic cleavage of the α-C−H bond of aminium radical cation **3** via PCET can yield iminium ion **5**. As shown in Figure 3, TEMPO-I and iodo radical as well as TEMPO and cationic TEMPO (TEMPO-C) were considered as the electron acceptors. As shown in Figure 5a, a TEMPO molecule starts the catalytic mechanism through single-electron transfer (SET) from the substrate, amino alcohol **1**. First, the substrate forms a loosely bound complex **7-a** that proceeds via a transition state (*) to form an adduct **7-b** at a relative solution-phase free energy of 14.55 kcal/mol. Complex **2** consumes 21.8 kcal/mol to produce aminium radical cation **3** and TEMPO-A. The 1H^+^/1e^−^ transfer (PCET) from aminium radical cation **3** to another TEMPO produces iminium **5** and TEMPO-H with −27.99 kcal/mol of solution-phase free energy, exhibiting a high stability. Intermediate **5** leads to a more stable and neutral oxyaminal **7** via a transition state **7-TS** of 27.99 kcal/mol above the starting complex. Finally, the resulting product **2** is readily formed by an exergonic reaction of −15.2 kcal/mol with a barrier of 14 kcal/mol. The PCET reaction can be theoretically explained by homolytic bond dissociation free energy (BDFE) [21]. The known BDFE from TEMPO-H to TEMPO is almost 65~70 kcal/mole according to the deviation of solvent system. It seems that the decrease in the free-energy gap (64.34 kcal/mol) between aminium radical cation **3** and iminium **5** can barely compensate the bond association energy from the second TEMPO to TEMPO-H.

Figure 5b shows that TEMPO-C, instead of free-radical TEMPO shown in Figure 5a, initiated the SET from the substrate, amino alcohol **1**. As expected, rather than the complex of substrate **1** and TEMPO (Figure 5a), the complex of substrate **1** and TEMPO-C (Figure 5b) showed very smaller ∆G (36.55 kcal/mol in Figure 5a vs. 5.11 kcal/mol in Figure 5b) between aminium radical cation **3** and the initial complex. As shown in Figure 5b, we also consider the formation of oxyaminal aminium radical cation **8** and the common key intermediate **5**. Radical cation **8** showed only 10.19 kcal/mol above **8-c**. Finally, the generation of product **2** showed 0.38 kcal/mol above the starting complex **1**. As shown in Figure 5c, the iodo radical accepted 1e^−^ from substrate **1**, producing aminium radical cation **3** with 5.11 kcal/mol above complex **9-a**. After obtaining iminium intermediate **5**, product **2** can be acquired through the more stable oxyaminal intermediate **7** than iminium **5**. As shown in Figure 5d, TEMPO-I initiated the reaction through ET, and aminium radical cation **3** can be produced with the increase of 5.11 kcal/mol above the starting complex.

### 4.2. Pathway B: α-Aminoalkyl Radical and HAT Mechanism

**Figure 6:**
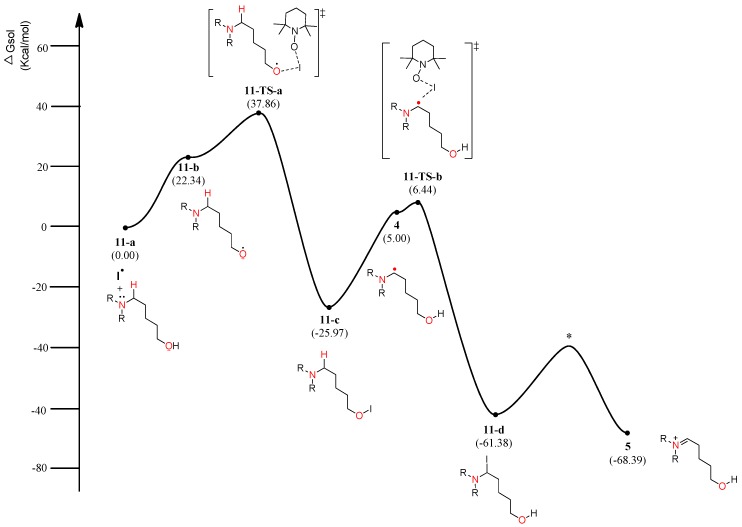
Calculated energy profile diagram for metal-free CDC reaction through pathway B; R-group is phenyl; transition state marked by asterisk (*) was not explicitly located.

The energy diagram of the second plausible mechanism was acquired, in which iminium intermediate **5** was produced from α-aminoalkyl radical **4** (Figure 6). As shown in the diagram, α-aminoalkyl radical **4** is generated through 1,6-H transfer followed by iodo radical elimination (“I–O” bond homolytic cleavage). TEMPO or iodo radical can be the initiators of the catalytic cycle. As shown in Figure 6, an iodo radical starts the catalytic mechanism through HAT (hydrogen-atom transfer) from the hydroxyl group in substrate **1**. O-radical **11-b** is located 22.34 kcal/mol above the starting complex **11-a**. The mechanism from O-radical **11-b** to 5-aminoalkyl hypoiodite **11-c** can be explained by **11-TS-a** located 15.52 kcal/mol above O-radical **2** and 63.83 kcal/mol below hypoiodite **11-c**. **11-TS-a** is similar to μ-oxo-bridged iodo derivatives with respect to the “I–O” bond length and angle of “O–I–O”. The stable and neutral hypoiodite **11-c** can lead to more stable α-iodo intermediate **11-d**. In the path, the location of α-aminoalkyl radical **4** is 17.34 kcal/mol below O-radical **11-b**. Although the solution-phase free energy of α-aminoalkyl radical **4** is close to the free energy of **11-TS-b** within a barrier of 1.5 kcal/mol, it is more stable than O-radical **11-b**. Through **11-TS-b**, α-aminoalkyl radical **4** can abstract an iodo radical from TEMPO-I generated from TEMPO and iodine (Figure 3). After α-iodo intermediate **11-d** is formed, the reaction proceeds via a transition state (*) and then forms the key intermediate **5** at a relative solution-phase free energy of −68.39 kcal/mol.

### 4.3. Pathway C: Conventional Ionic Mechanism

**Figure 7:**
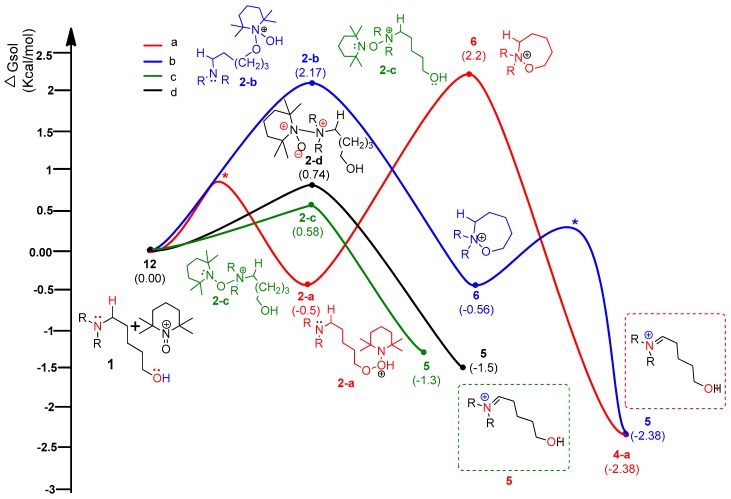
Calculated energy profile diagram for metal-free CDC reaction through pathway **C** (ionic mechanism); R-group is phenyl; transition state marked by asterisk (*) was not explicitly located.

According to the assumed four types of routes, the energy diagram of the ionic mechanism was acquired. All the catalytic cycles were proposed to be started from the TEMPO-C approaching substrate, amino alcohol **1**. Each color in Figure 7 indicates four types of routes with four types of initial intermediates after reacting with TEMPO-C. Path **C-a** (A), as shown in red color, is initiated by the approach of TEMPO-C to yield **2-a** with a free energy of −0.5 kcal/mol via an uncalculated transition state, described with the symbol *. The intramolecular cyclization of intermediate **2-a** gives the unstable oxazepanium intermediate **6** with the “O–O” bond breakage and “N–O” bond formation, and the overall barrier for the step was calculated to be 1.7 kcal/mol. Then, the oxidative elimination of α-proton produces the iminium key intermediate **5** located 4.58 kcal/mol below oxazepanium **6**. Finally, the intramolecular cyclization of the resulting intermediate **5** to 2-amino tetrahydropyran **2** was omitted in the calculation. In path C-b (B), the starting complex **1** generates intermediate **2-b** through the “N–O” bond formation between cationic TEMPO and substrate **1** rather than the “O–O” bond formation between them in path C-a. The calculated barrier of 2.17 kcal/mol of **2-b** showed that intermediate **2-b** is more favored than intermediate **2-a**. The barrier energy for **2-b** due to a high stearic hindrance is supposed to present the backward reaction of **2-b** to complex **1**. The proposed paths **C-c** and **C-d** also show the intermediates **2-c** and **2-d** as being less stable than **2-a**, but after their formation, the paths proceed under a lower free energy level than paths **C-a** or **C-b**. Therefore, it is assumed that path **C-a** is favored to form the first intermediate. In the thermodynamic equilibrium, the existing ratio of **2-a** is the largest among the four intermediates (**2-a**, **2-b**, **2-c**, and **2-d**), but the forward reaction to the iminium key intermediate **5** favors path **C-c**.

### 4.4. Combined Analysis of Mechanistic Study

**Figure 8:**
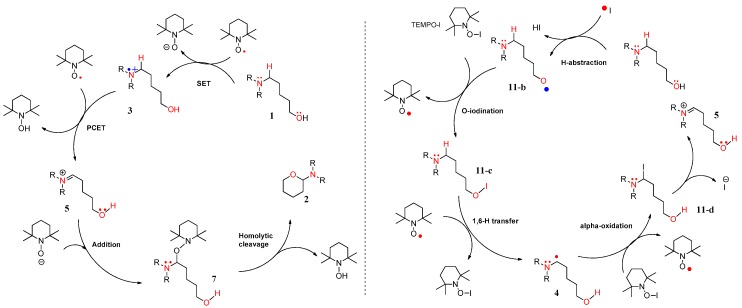
Plausible nonionic mechanisms for metal-free CDC reaction of compound **1**: pathways A and B.

From the analysis of four types of path A, we propose that iminium intermediate **5** can be converted to slightly more stable oxyaminal intermediate **7** resulting from the addition of TEMPO to iminium **5**. Among the four types of reactive species (TEMPO, TEMPO-C, TEMPO-I, and iodo radical), despite a comparatively high energy transition state, the TEMPO-initiated cyclization shows the largest free-energy gap between the starting complex and product **2**. The following results of the commonly-proposed plausible routes are neither surprising nor unexpected. The TEMPO-initiated mechanism is described in Figure 8 (left side). In the mechanism, two TEMPO molecules produced two TEMPO-Hs after the dehydrogenative coupling, and a TEMPO radical can be regenerated under the BDFE from TEMPO-H to TEMPO. In the case of path B, similar to the modified Suárez hypoiodite oxidation, the 1,6-H transfer in 5-aminoalkyl hypoiodite **11-c** affords α-aminoalkyl radical **4** with an increase of ~30 kcal/mol [31]. Although examples for the formation of aminoalkyl radicals by HAT are limited in photoredox catalysis [32], the stabilizing effect produced by the *N,N*-diarylamino group prompted us to propose the formation of α-aminoalkyl radical **4** and the sequential formation of α-iodo intermediate **11-d** showed −61.38 kcal/mol below the starting complex to support the possibility of the pathway. The overall concrete pathway is shown in Figure 8 (right side). In addition, when TEMPO was replaced with a stable aminium radical cation (TBPA; tris-4-bromophenyl ammonium hexachloroantimonate), a reported initiator of α-aminoalkyl radical, the corresponding cyclization product was observed in our synthetic experiment [9]. When considering three key intermediates (aminium radical cation **3**, α-aminoalkyl radical **4**, and iminium **5**), cation **3** was more stable than our expectation due to the hyperconjugation of substituted aromatic rings, and the free energy of iminium **5** explains why we did not observe the intermediate at all. When comparing three paths, it seems that the ionic pathway (path C) is more favored in the view of the free energy of TS, but cannot exclude the possibility of two single-electron oxidations. In particular, the free-energy gap between a starting complex and the key intermediate iminium **5** was the highest in path **B**.

## 5. Conclusions

In summary, the TEMPO/I_2_-mediated oxidative cyclization of 1,5-amino alcohol 1 as a metal-free CDC reaction was studied using DFT-calculated energy diagrams. To get the diagrams, the three pathways (SET/PCET mechanism, HAT/1,6-H transfer mechanism, and ionic mechanism), three key intermediates (aminium radical cation **3**, α-aminoalkyl radical **4**, and iminium **5**), and four reactive species (radical TEMPO, cationic TEMPO, TEMPO-I, and iodo radical). As general expectations, TEMPO was more favored than TEMPO-I or iodo radical in SET/PCET pathway. With the evidence of our catalytic radical reaction, the iodo-radical-initiated HAT pathway showed a larger free-energy gap between the starting complex and iminium intermediate 5. The free energy of TS was the lowest in the ionic pathway. In the future, we hope to develop metal-free CDC methodologies for other substrates based on the understanding acquired in this study. In particular, we hope from the mechanistic study to get an idea for designing in situ generated new iodo reagents.

## Abbreviations

CDC	Cross-dehydrogenative coupling
PCET	Proton-coupled electron transfer
SET	Single-electron transfer
HAT	Hydrogen-atom transfer
GEA	Electron attachment free energy
SCF	Self-consistent field
ZPE	Zero-point energy
BDFE	Homolytic bond dissociation free energy
